# First palaeoneurological study of a sauropod dinosaur from France and its phylogenetic significance

**DOI:** 10.7717/peerj.7991

**Published:** 2019-11-18

**Authors:** Fabien Knoll, Stephan Lautenschlager, Xavier Valentin, Verónica Díez Díaz, Xabier Pereda Suberbiola, Géraldine Garcia

**Affiliations:** 1 ARAID-Fundación Conjunto Paleontológico de Teruel-Dinópolis, Teruel, Spain; 2 Departamento de Paleobiología, Museo Nacional de Ciencias Naturales-CSIC, Madrid, Spain; 3 School of Geography, Earth and Environmental Sciences, University of Birmingham, Birmingham, UK; 4 Laboratoire de Paléontologie, Evolution, Paléoécosystèmes et Paléoprimatologie, Université de Poitiers, Poitiers, France; 5 Museum für Naturkunde, Leibniz-Institut für Evolutions-und Biodiversitätsforschung, Berlin, Germany; 6 Humboldt Universität zu Berlin, Berlin, Germany; 7 Departamento de Estratigrafía y Paleontología, Universidad del País Vasco/Euskal Herriko Unibertsitatea, Bilbao, Spain

**Keywords:** Titanosauria, Palaeoneurology, Cretaceous, Phylogeny, France

## Abstract

Despite continuous improvements, our knowledge of the palaeoneurology of sauropod dinosaurs is still deficient. This holds true even for Titanosauria, which is a particularly speciose clade of sauropods with representatives known from numerous Cretaceous sites in many countries on all continents. The data currently available regarding the palaeoneurology of titanosaurs is strongly biased towards Gondwanan forms (Argentina above all, but also India, Malawi and Australia). In contrast, the palaeoneurology of Laurasian titanosaurs is known only from a few taxa from Spain and Uzbekistan, despite the discovery in other countries of Laurasia of a number of neurocranial remains that would lend themselves well to investigations of this kind. To fill in this gap in our knowledge, we subjected a titanosaurian braincase from the uppermost Upper Cretaceous of southern France to X-ray computed tomographic scanning, allowing the generation of 3D renderings of the endocranial cavity enclosing the brain, cranial nerves and blood vessels, as well as the labyrinth of the inner ear. These reconstructions are used to clarify the phylogenetic position of the specimen from the Fox-Amphoux-Métisson site. A combination of characters, including the presence of two hypoglossal rami on the endocast, the average degree of development of the dorsal-head/caudal-middle-cerebral vein system and the relatively short and subequal lengths of the ipsilateral semicircular canals of the labyrinth, are particularly revealing in this respect. They suggest that, compared with the few other Laurasian titanosaurs for which in-depth palaeoneurological data are available, the French taxon is more derived than the distinctly more ancient, possibly non-lithostrotian titanosaur from the Uzbek site of Dzharakuduk but more basal than derived saltasaurids, such as the coeval or slightly more recent forms from the Spanish locality of Lo Hueco.

## Introduction

The first observations on the palaeoneurology of sauropod dinosaurs were made almost 140 years ago (Marsh, 1880: unnumb. fig.). Nevertheless, the group has since been the focus of relatively scanty investigations on a few species (see in particular Witmer et al., 2008, and references therein; Paulina Carabajal, Coria & Chiappe, 2008; Knoll & Schwarz-Wings, 2009; Wilson et al., 2009; Balanoff, Bever & Ikejiri, 2010; Knoll et al., 2012, 2013, 2015; Paulina Carabajal, 2012; Paulina Carabajal, Carballido & Currie, 2014; Sues et al., 2015; Martínez et al., 2016; Paulina Carabajal, Canale & Haluza, 2016; Bronzati, Benson & Rauhut, 2018; Paulina Carabajal et al., 2018). This is, in part, due to the fact that braincases of sauropods are extremely rare. Sauropods are especially interesting to the palaeoneurologist because of their *Bauplan*, which features, at the extremity of a variously elongated neck, a head of conspicuously small size relative to the generally imposing body (Sander et al., 2011; Hallett & Wedel, 2016). The size of the body reaches the maximum known in any land tetrapod within a speciose clade of Cretaceous sauropods, the Titanosauria (see e.g., Benson et al., 2014). Over the last decade, our knowledge of the palaeoneurology of titanosaurian sauropods has dramatically improved. While the endocranial morphology of titanosaurs has long been known only in *Jainosaurus septentrionalis* (Huene & Matley, 1933) from the Maastrichtian of India, a considerable amount of new data on the endocranial and inner ear morphology in taxa from Argentina, Spain, India, Uzbekistan, Australia and Malawi have recently been gathered (Paulina Carabajal, 2012; Knoll et al., 2013, 2015; Paulina Carabajal, Filippi & Knoll, 2015; Sues et al., 2015; Martínez et al., 2016; Poropat et al., 2016; Andrzejewski et al., 2019). However, this represents only a diminutive fraction of titanosaurian taxic diversity. In fact, while the descriptions of new titanosaur species are regularly published, they rarely include any neurocranial remains (Wilson et al., 2016; for recently described titanosaurs lacking braincase material see e.g., Calvo & Gonzalez Riga, 2019; Gorscak & O’Connor, 2019). Nevertheless, a number of titanosaurian braincases have yet to be examined from a palaeoneurological viewpoint (through modern imaging technologies or otherwise). This holds especially true for the Upper Cretaceous of Europe. Europeans specimens represent significant data to help assess the diversity of titanosaurs that are being neglected. The aim of the present article is to describe the digital endocast of an isolated braincase of an unnamed titanosaur from the Upper Cretaceous of the Provence-Alpes-Côte d’Azur region of southern France and to build on this reconstruction to clarify the phylogenetic position of the specimen.

## Materials and Methods

The present study focuses on a reconstruction of the endocranial and labyrinthine cavities of a previously described, but undiagnosed braincase, FAM 03.064, from the Campanian locality of Fox-Amphoux-Métisson (Montmeyan syncline), Var, southeastern France (Díez Díaz et al., 2012). The specimen comes from the “Grès à Reptiles” Formation and more specifically from a sandstone deposit yielding wood, invertebrates and a variety of vertebrates (selachians, bony fishes, turtles, squamates, crocodilians and dinosaurs). The dinosaur fauna itself is diverse (theropods, sauropods, ankylosaurs and ornithopods), although only sauropod dental and cranial specimens have been examined in detail so far (Díez Díaz et al., 2012).

It was identified as belonging to Titanosauria on the basis of a combination of characters purportedly not found outside this clade (including a pair of well-defined crescentic transverse nuchal crests on the parietal and other features involving different neurocranial bones; see Díez Díaz et al., 2012: p. 635). Its precise affinities with other titanosaurs could not be clarified, except for the fact that several aspects of its morphology set it apart from other European titanosaurian braincases (Díez Díaz et al., 2012: p. 635), all of which either Campanian or Maastrichtian in age.

To produce a three-dimensional reconstruction of the endocast of the cranial cavity and endosseous labyrinth of the inner ear, the specimen was subjected to computed tomographic (CT) scanning at the Université de Poitiers (Poitiers, France), using a EasyTom XL duo (RX-Solutions, Chavanod, France) with a voltage of 130 kV and a current of 440 μA. The dataset consisted of 1165 slices (1089 × 1034 × 1165 pixels, 0.116 mm voxel size) in tiff format.

**Figure 1:**
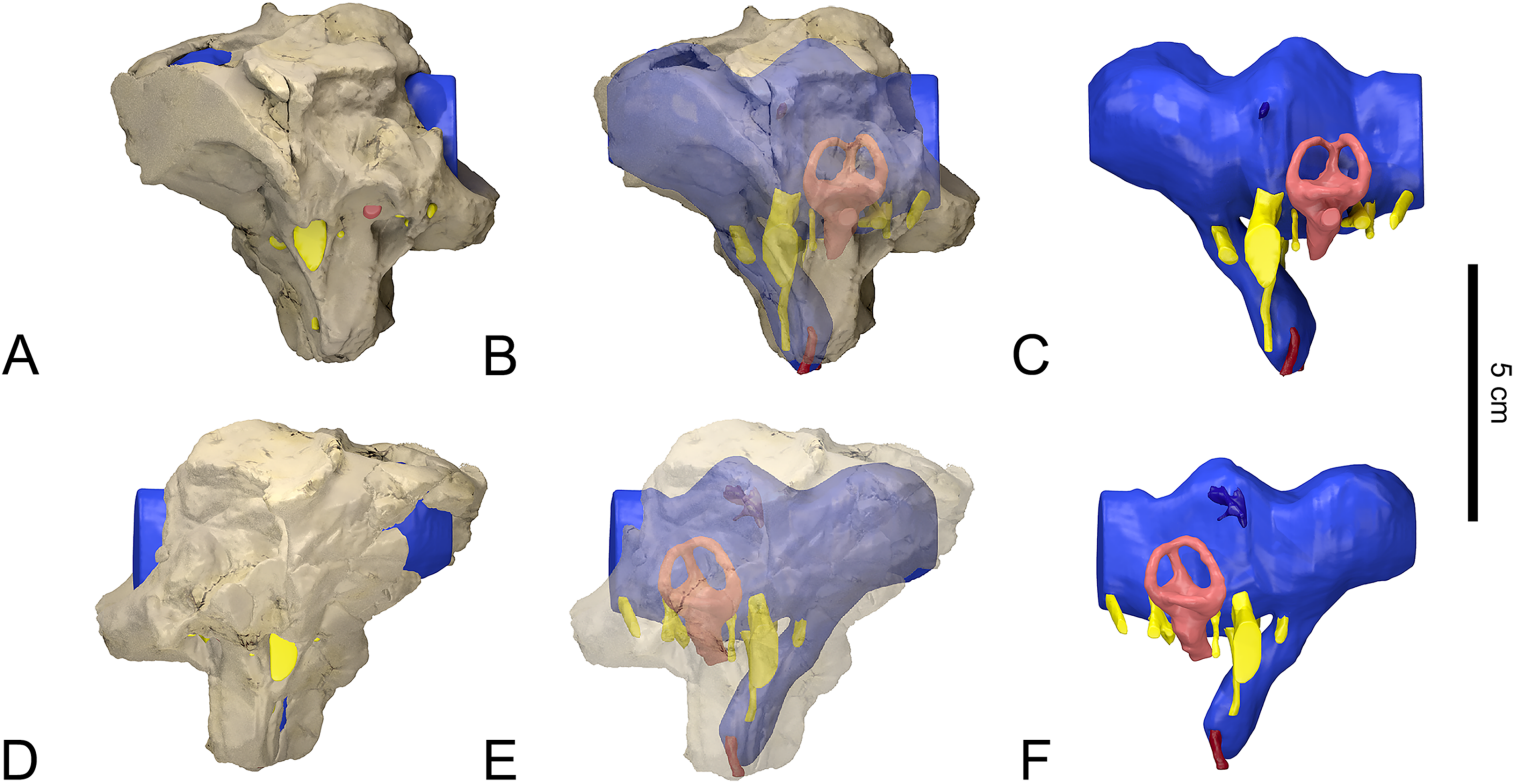
Surface-rendered CT-based reconstructions of the braincase, cranial endocast and associated soft tissues structures of the indeterminate titanosaurian specimen (FAM 03.064) from the Late Cretaceous of Fox-Amphoux-Métisson, France. Bone rendered opaque (A, D), made semitransparent (B, E) and removed (C, F). In left (A–C) and right (D–F) lateral views.

**Figure 2:**
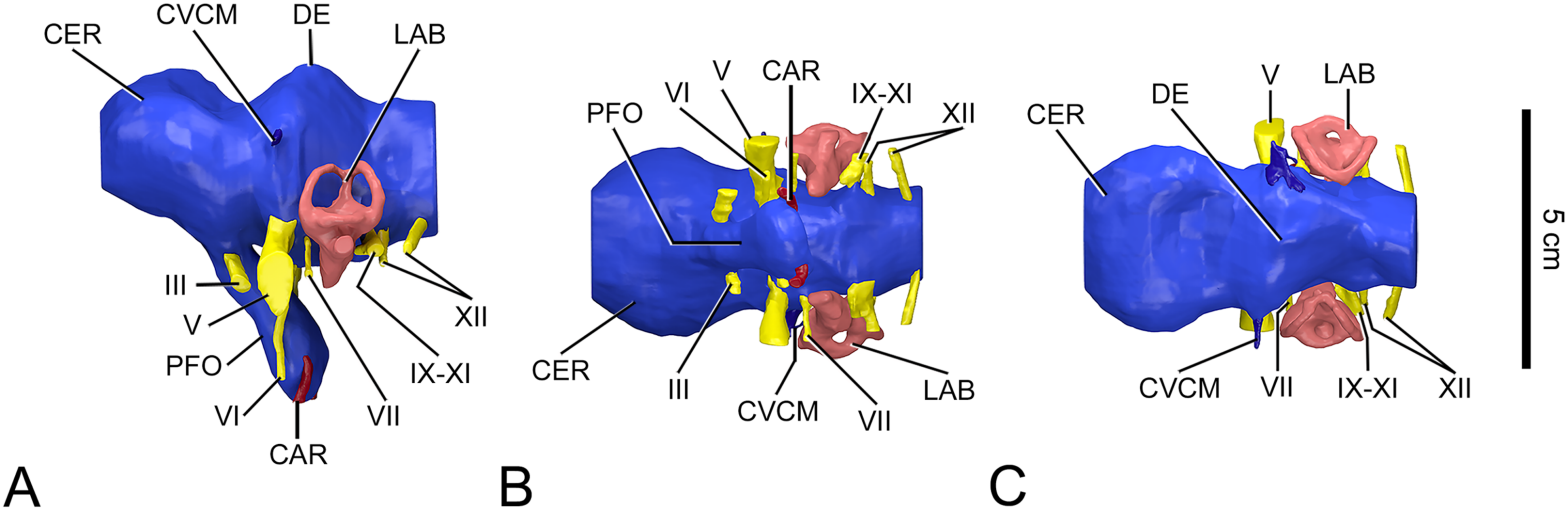
Surface-rendered CT-based reconstructions of the cranial endocast and associated soft tissues structures of the indeterminate titanosaurian braincase (FAM 03.064) from the Late Cretaceous of Fox-Amphoux-Métisson, France. In lateral (A), ventral (B) and dorsal (C) views. Abbreviations: III, oculomotor nerve; V, trigeminal nerve; VI, abducens nerve; VII, facial nerve; IX-XI, glossopharyngeal and vagoaccessory nerves; XII, hypoglossal nerve; CAR, internal carotid artery; CER, cerebrum; CVCM, caudal middle cerebral vein; DE, dural expansion; LAB, labyrinth; PFO, pituitary body.

**Figure 3:**
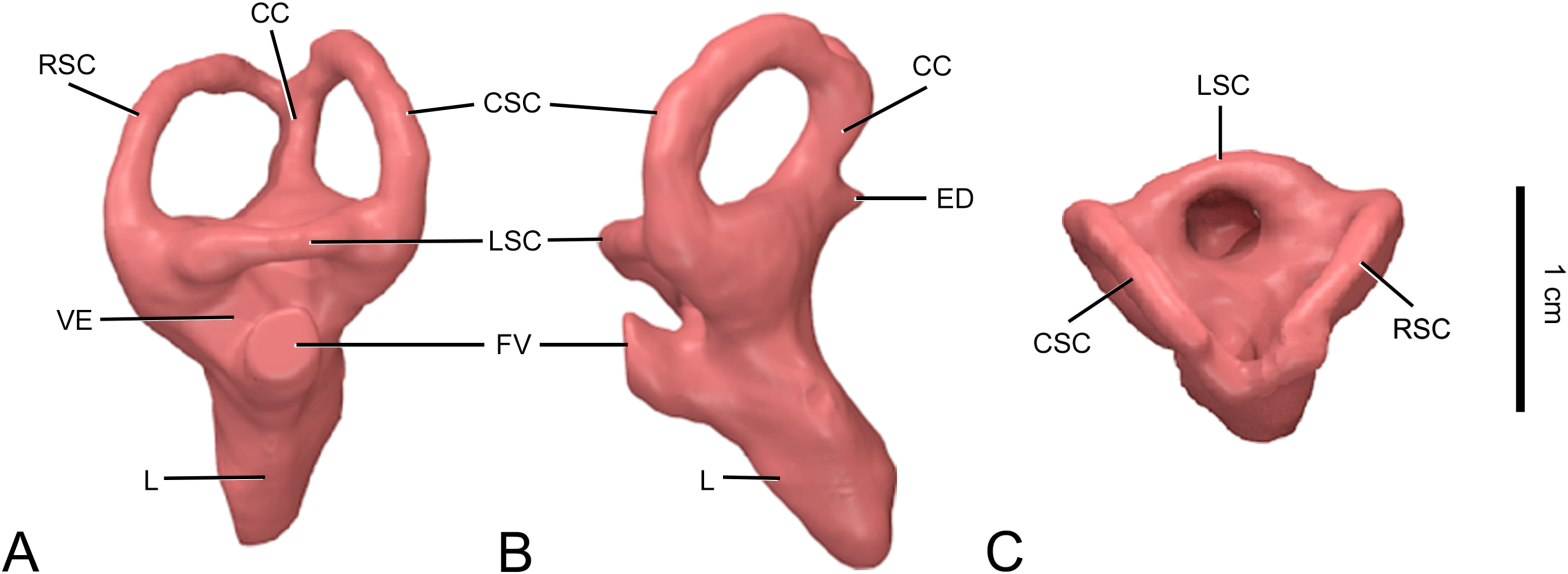
Surface-rendered CT-based reconstruction of the left endosseous labyrinth of the indeterminate titanosaurian braincase (FAM 03.064) from the Late Cretaceous of Fox-Amphoux-Métisson, France. In lateral (A), caudal (B) and dorsal (C) views. Abbreviations: CC, common crus; CSC, caudal semicircular canal; ED, endolymphatic duct; FV, oval window; L, lagena; LSC, lateral semicircular canal; RSC, rostral semicircular canal; VE, vestibule.

The CT data were imported into Avizo 7.0 (Visualisation Science Group, Burlington, MA, USA) for image segmentation and digital reconstruction. Anatomical structures of interest (endocasts, endosseous labyrinths, neurovascular structures) were labelled using Avizo’s segmentation editor. The magic wand tool was used where possible to perform the segmentation semi-automatically (with thresholds for bone set to a greyscale value larger than 35,000, a value of 25,000–35,000 for sediment infill and values below 5,000 for air-filled cavities). In regions with poor contrast between matrix, bone and structures of interest the paintbrush tool was used for manual segmentation. 3D surface models and volumes were created to visualize the segmented structures and to illustrate this article with traditional figures (Figs. 1–3). In addition, surface models of the individual structures were downsampled to a degree that allowed for small file sizes, but preserved all details, and were exported as separate OBJ files for the creation of the interactive 3D model of Fig. S1, as outlined in Lautenschlager (2014: pp. 116-117) using Adobe 3D reviewer (Adobe Systems Inc., San Jose, CA, USA).

The palaeoneurology of FAM 03.064 is compared below with that of the other titanosaurs from the Late Cretaceous of Eurasia and India for which endocasts (be they physical or digital) of the cranial cavity and endosseous labyrinth have been described (Table 1) as well as with species from different times and locations when appropriate.

**Table 1:** Titanosaurian endocasts used for comparison in this study.

Specimen	Taxon	Age	Country
GSI K27/497 (Huene & Matley, 1933: fig. 6; Wilson et al., 2009: fig. 7)	*Jainosaurus septentrionalis* (Huene et Matley, 1933)	Maastrichtian	India
MPCM-HUE-8741 (Knoll et al., 2013: figs 3, 4, S1, S2, S3)	*Lohuecotitan pandafilandi* (Díez Díaz et al., 2016)	Campanian	Spain
MPCM-HUE-1667 (Knoll et al., 2015: figs 4, 5, 7, S1)	Lithostrotia indet.	Campanian	Spain
CCMGE 628/12457 (Sues et al., 2015: figs 3, 4, Witmer, 2019)	Titanosauria indet.	Turonian	Uzbekistan

## Results

### Systematic palaeontology

Dinosauria Owen, 1842
Saurischia Seeley, 1888
Sauropoda Marsh, 1878
Titanosauria Bonaparte et Coria, 1993
Titanosauria indet.

### Description and comparison

As the median part of the conjoined frontals has broken away in FAM 03.064, the top of the cerebrocast had to be inferred to a degree. The same holds true for the ventral side of the endocast due to the incomplete state of preservation of the orbitosphenoids (the left rostrolateral region of the cerebrocast is mirrored from the right side, which could be reconstructed). Apart from these minor aspects, the CT scan data permitted a comprehensive rendering of the cranial endocast and endosseous labyrinths (Figs. 1–3). Most cranial nerves, as well as major vascular structures, could be reconstructed on both sides. Reconstructions of both the right and left labyrinths could also be achieved.

#### Brain endocast

The incompleteness of FAM 03.064 rostrodorsally makes it impossible to gauge the length of the olfactory tract (Fig. 1). In this regard, the specimen is similar to others. For example, the state of GSI K27/497 prevents making appropriate observations regarding the olfactory tracts and bulbs in *J. septentrionalis* (Wilson et al., 2009: fig. 7). The condition of CCMGE 628/12457 is worse still, as both frontals seem to be missing entirely in this specimen of indeterminate titanosaur (Sues et al., 2015: figs 2C; Witmer, 2019). In contrast, the frontals of MPCM-HUE-8741, a specimen that is here tentatively attributed to *Lohuecotitan pandafilandi*, are well preserved. They cover very short olfactory tracts rostrally (Knoll et al., 2013: figs 3A, C, D, S1, S2, S3). In MPCM-HUE-1667, an unnamed lithostrotian titanosaur, the olfactory tracts could be reconstructed in their entirety. They are extremely short (Knoll et al., 2015: figs 4, 5C, D, S1). This is consistent with shortened nasal bones and, therefore, external nares with greatly retracted caudal margin as observed in other titanosaurs (Curry Rogers & Forster, 2004; Wilson, 2005; Martínez et al., 2016; Wilson et al., 2016) and indeed many other sauropods.

As in other titanosaurs, especially derived forms such as MPCM-HUE-1667 (Knoll et al., 2015: figs 4, S1), the endocast of FAM 03.064 appears fairly straight in lateral view (Fig. 1). This stands in sharp contrast with the endocast of the basal titanosauriform *Giraffatitan brancai*, the flexures of which make it look more clearly sigmoid in lateral view (Knoll & Schwarz-Wings, 2009: figs 1, 2A, B). In fact, an endocast that appears strongly sigmoid in lateral view is the plesiomorphic condition in sauropodomorphs (see e.g., Sereno et al., 2007: fig. 1G). The endocast of FAM 03.064 is lageniform in outline in dorsal view (Fig. 2C), strongly recalling the homologous portions of the endocasts of both *L. pandafilandi* (Knoll et al., 2013: figs 3C, S1, S2, S3) and MPCM-HUE-1667 (Knoll et al., 2015: fig. 5D, S1) in that respect. The cerebrocast is marked by a caudomedian depression dorsally, which gives it a C-shaped aspect in dorsal view (Fig. 2C). A roughly similar configuration is seen in *L. pandafilandi* (Knoll et al., 2013: figs 3C, S1, S2, S3), which was interpreted as being due to paired longitudinal dural venous sinuses coursing dorsolaterally over the cerebral region (Knoll et al., 2013: p. 7). The cerebrocast constitutes the widest portion of the endocast. The forebrain region is separated from the midbrain-hindbrain complex by a marked constriction (Figs. 1 and 2), which is caused by a sturdy internal laterosphenoid pillar. A second medial narrowing affects the caudal quarter of the endocast (Figs. 1 and 2). It corresponds to the space occupied by the otic capsule within the lateral wall of the braincase. A tiny knob dorsal to the facial nerve on the right side of the endocast (best observable in Fig. S1 when the right labyrinth is unticked) may be due to a diminutive fossa auriculae cerebelli (which in life housed the cerebellar flocculus; see e.g., Ferreira-Cardoso et al., 2017). This is the only tangible indication of the cerebellum. No visible relief on the endocast is present in *J. septentrionalis* (Wilson et al., 2009: fig. 7A), *L. pandafilandi* (Knoll et al., 2013: figs 3A, S1, S2, S3), MPCM-HUE-1667 (Knoll et al., 2015: figs 4, S1) or CCMGE 628/12457 (Sues et al., 2015: fig. 3A). In fact, embossments related to the cerebellar flocculus are rare in sauropod endocasts. They are best developed in *G. brancai* (Knoll & Schwarz-Wings, 2009: figs 1, 2A–D), in which they lodge themselves in small but sharply delimited dents on the medial surface of the prootic (Janensch, 1935–1936: fig. 116). In basalmost sauropodomorphs (see e.g., Bronzati et al., 2017: fig. 2), the floccular fossae may be more developed than in more derived forms.

Despite the poor preservation of the cranial vault in the forebrain region in FAM 03.064, it can be determined that this part of the brain was not covered by a large, irregularly-shaped dural expansion (Figs. 1, 2A and 2C). This is similar to what can be observed on the endocasts of both Lo Hueco lithostrotians (Knoll et al., 2013: figs 3A, C, S1, S2, S3, Knoll et al., 2015: figs 4, 5A, D, S1), which lack a voluminous rostral dural expansion, but this is in contrast with the situation in a number of other sauropods, such as *G. brancai* (Knoll & Schwarz-Wings, 2009: figs 1, 2A, B) and the putative basal lithostrotian *Sarmientosaurus musacchioi* (Martínez et al., 2016: figs 9A, D, S1), for instance, whose endocasts show crag-like enlargements that, presumably, are indicative of a sizeable venous sinus. The hindbrain of FAM 03.064 appears to support a relatively large caudal dural expansion (Figs. 1, 2A and 2C), which may be due to a confluence between the transverse (“middle cerebral vein”), dorsal sagittal and occipital sinuses. A very similar configuration is found in MPCM-HUE-1667 (Knoll et al., 2015: figs 4, 5A, B, D, S1) and the endocast of *J. septentrionalis* (Wilson et al., 2009: fig. 7A) is comparable in this respect. In its plain rather than complicated shape, this caudal dural expansion differs from those in CCMGE 628/12457 (Sues et al., 2015: figs 3A, B, D, E), *S. musacchioi* (Martínez et al., 2016: figs 9A, B, D, S1), *G. brancai* (Knoll & Schwarz-Wings, 2009: figs 1, 2A, B) and a number of more basal sauropods. The caudal half of the endocast of *L. pandafilandi* does not display any large caudal dural expansion, but only a minor convoluted protrusion (Knoll et al., 2013: figs 3A–C, S1, S2, S3). All in all, FAM 03.064 resembles MPCM-HUE-1667 (Knoll et al., 2015: figs 4, 5) and *J. septentrionalis* (Wilson et al., 2009: fig. 7) closely, as far as the overall morphology of the endocast is concerned.

As in most sauropodomorphs (Witmer et al., 2008), the endocast of FAM 03.064 exposes a large hypophysis (pituitary) that extends caudoventrally (Figs. 1, 2A and 2B). Similar to *G. brancai* (Knoll & Schwarz-Wings, 2009: figs 1, 2A, B) but in contrast with most other sauropods (see e.g., Witmer et al., 2008: figs 6.8, 6.9; Balanoff, Bever & Ikejiri, 2010: fig. 10), the infundibulum is not constricted (Fig. 1). The hypophysis is claviform in rostral or caudal view but rather musiform (with the concavity situated rostrally) in lateral view (Fig. 1). It is the widest at the level of the entrance of the internal carotid arteries. The long axis of the hypophysis makes an angle of about 65° with that of the lateral semicircular canal (Fig. 1). This is more obtuse than in *L. pandafilandi* (~55°; Knoll et al., 2013: figs 3A, S1, S2, S3), MPCM-HUE-1667 (~45°; Knoll et al., 2015: figs 4, S1) and also, although to a lesser degree, CCMGE 628/12457 and *Malawisaurus dixeyi* (~60°; Sues et al., 2015: fig. 3A; Andrzejewski et al., 2019: figs 2A, S1). This angle is also about 65° in the Lithostrotia *incertae sedis Bonatitan reigi* (Paulina Carabajal, 2012: fig. 2A), not far off the 70° observed in *G. brancai* (Knoll & Schwarz-Wings, 2009: figs 1, 2A, B). The morphology of the hypophysis in the endocast of FAM 03.064 is very different from that in CCMGE 628/12457 (Sues et al., 2015: fig. 3A, B, C, E), which is composed of a slender but humpbacked proximal half and a bulbous distal half. In contrast, it compares well with the hypophysis of MPCM-HUE-1667 (Knoll et al., 2015: figs 4, 5A, B, C, S1). However, in the latter the caudoventral orientation of the hypophyseal body is attained through a bend of the infundibular stalk (as in *L. pandafilandi*; Knoll et al., 2013: figs 3A, S1, S2, S3), whereas in FAM 03.064 the infundibulum is in line with the rest of the hypophysis (Fig. 1; like in CCMGE 628/12457; Sues et al., 2015: fig. 3A).

#### Cranial nerves

Neither orbitosphenoid is complete enough in FAM 03.064 to allow reconstructing the optic nerve (CN II).

The oculomotor nerve (CN III) emerges from the endocast in the lateral surface of the infundibulum (Figs. 2A and 2B). It then courses ventrolaterally to leave the braincase through the suture between the orbitosphenoid (rostrally) and the laterosphenoid (caudally), that is in the orbit. The oculomotor nerve is inadequately known in *J. septentrionalis* (Wilson et al., 2009), but there does not appear to be any meaningful differences in this respect between FAM 03.064 and *L. pandafilandi* (Knoll et al., 2013: figs 3A, D, S1, S2, S3), MPCM-HUE-1667 (Knoll et al., 2015: figs 4, 5A, C, S1) and CCMGE 628/12457 (Sues et al., 2015: figs 3A, C, E, Witmer, 2019).

The trochlear nerve (CN IV) could not be reconstructed in FAM 03.064. It is possibly represented on the lateral wall of the braincase by only a tiny foramen on the rostral margin of the laterosphenoid (Díez Díaz et al., 2012: fig. 5B, E, F), but the resolution of the CT data allowed no unambiguous reconstruction of the nerve canal. If it could have been traced digitally, it may have looked fairly similar in diameter and location to the trochlear nerve in MPCM-HUE-1667 (Knoll et al., 2015: figs 3D, S1).

The trigeminal nerve (CN V) is, as expected, the biggest of the cranial nerves (Fig. 2). Its largest diameter is much greater than that of the metotic group (CN IX-XI; vide infra). In cross section, the trigeminal nerve forms an ellipse with a dorsoventrally oriented major axis all along its ventrolateral course from the endocast to the external foramen on the laterosphenoid-prootic suture. So, the in vivo division of this nerve into rostral (ophthalmic, CN V_1_) and caudal (maxillomandibular, CN V_2,3_) rami is not patent osteologically (even though the trigeminal foramen does have a somewhat cordiform rim laterally). The configurations of the trigeminal nerve in FAM 03.064 and MPCM-HUE-1667 (Knoll et al., 2015: figs 4, 5A, C, D, S1) are similar and no relevant difference with CCMGE 628/12457 (Sues et al., 2015: figs 3A, C, E; Witmer, 2019) can be detected in this regard. In *L. pandafilandi* (Knoll et al., 2013: figs 3, S1, S2, S3), the intraosseous path of the trigeminal nerve appears shorter as it is directed more laterally. Little is known about the trigeminal nerve in *J. septentrionalis* (Wilson et al., 2009).

The abducens nerve (CN VI) emerges ventrally from the pontine portion of the brainstem, ventral to the trigeminal nerve (Figs. 2A and 2B). It then courses rostroventrally, gets past the pituitary body laterally, very closely (a solid but thin wall of bone separates them), and leaves the braincase from the rostral surface of the parabasisphenoid (Fig. 1; see also Díez Díaz et al., 2012: figs 3A, 4B, C). As far as the state of preservation permits to determine, the configuration was similar in *L. pandafilandi* (Knoll et al., 2013: figs 3B, D, S1, S2, S3). The abducens nerves either penetrate the pituitary fossa (MB.R.2180.22.1-4, M.B.R.2384) or pass in its periphery (MB.R.2223.1) in *G. brancai* (Janensch, 1935–1936: p. 259, figs 117, 118, pl. 13 fig. 1a; Knoll & Schwarz-Wings, 2009: figs 1, 2A, B; F Knoll, 2009, personal observations). In *Europasaurus holgeri*, another basal titanosauriform, the abducens nerves pierce the floor of the endocranial cavity close to one another, but then diverge so that they do not enter the pituitary fossa (A Schmitt, F Knoll & E Tschopp, 2015, personal observations). In most sauropods, the abducens canal cohere with the pituitary fossa (see e.g., Witmer et al., 2008; Knoll et al., 2012; Paulina Carabajal, Carballido & Currie, 2014). Whereas the abducens nerve flanks the hypophysis closely in FAM 03.064, it passes at bay in MPCM-HUE-1667 (Knoll et al., 2015: figs 5A, B, C, S1).

The facial nerve (CN VII) emerges from the brainstem about halfway between the trigeminal nerve (CN V) and the inner ear region (Figs. 2A and 2B), close to the vestibulocochlear nerve (CN VIII). It orientates ventrolaterally in parallel with the trigeminal nerve. After running within the prootic, the facial nerve leaves the braincase on the caudal side of the prootic crest. Externally, a main branch of it diverges to run ventrally on the prootic and then on the parabasisphenoid lateral surface, as a well-defined, fairly straight channel in the rostroventral sector of the middle ear recess demonstrates (see Díez Díaz et al., 2012: figs 4, 5E, F). The intraosseous route of the facial nerve is similar in FAM 03.064, MPCM-HUE-1667 (Knoll et al., 2015: figs 5A, C, S1) and CCMGE 628/12457 (Sues et al., 2015: figs 3A, C; Witmer, 2019). Unfortunately, the course of the facial nerve is not well known in *L. pandafilandi* (Knoll et al., 2013: figs 3D, S1, S2, S3), nor is it in *J. septentrionalis* (Wilson et al., 2009: fig. 7A). No channel for the facial nerve is present on the external surface of the braincases of either *L. pandafilandi* (Knoll et al., 2013: figs S1, S2, S3), CCMGE 628/12457 (Sues et al., 2015: figs 1A, C, 2A; Witmer, 2019) or, as it seems, *J. septentrionalis* (GSI K27/497; Wilson et al., 2009: fig. 4A). In contrast, a channel, functionally equivalent but not so distinct structurally, is present on MPCM-HUE-1667 (Knoll et al., 2015: figs 3C, S1).

Whereas the cochlear branch of the vestibulocochlear nerve (CN VIII) could not be traced in the FAM 03.064 CT scan data, the vestibular one could be visualized as it innervates the vestibular labyrinth in the region of the ampulla of the rostral semicircular canal (Fig. S1). No part of the vestibulocochlear nerve could be reconstructed in *L. pandafilandi* (Knoll et al., 2013), and no information is available either in this respect in *J. septentrionalis* (Wilson et al., 2009). In contrast, this zone could be segmented to the same limited extent as in FAM 03.064 in MPCM-HUE-1667 (Knoll et al., 2015: fig. S1). The configuration is similar in these two specimens, with a short vestibular branch of the vestibulocochlear nerve connected with the ampulla of the rostral semicircular canal. Connections of the inner ear with the endocranial space have been rendered in exceptional quality in CCMGE 628/12457 (Sues et al., 2015; Witmer, 2019). Thus, CCMGE 628/12457 shows an additional division of the vestibular branch of the vestibulocochlear nerve compared with FAM 03.064 (as well as the endolymphatic duct). This supplementary division of the vestibular branch could be segmented also in *S. musacchioi* (Martínez et al., 2016: fig. S1), in which it is spatially closer to that innervating the ampulla of the rostral semicircular canal than in CCMGE 628/12457.

The glossopharyngeal and vagoaccesory nerves (CN IX-XI) are combined together and with other structures into a metotic group situated between the labyrinth of the inner ear rostrally and the rostral hypoglossal branch caudally (Figs. 2A and 2B). The metotic group extends ventrolaterally, filling out the caudal part of the metotic cavity (cavum metoticum), which is cordiform in outline in ventrolateral view of the braincase. This caudal lobe of the metotic cavity accommodates the metotic foramen, whereas the rostral lobe, of smaller size, houses the oval window. As far as preservation allows the observation of this region, the configuration seems to have been similar in *L. pandafilandi* (Knoll et al., 2013: figs S1, S2, S3). In contrast, the metotic foramen and oval window are not confined into a hollow of heart-shaped contour in MPCM-HUE-1667 (Knoll et al., 2015: fig. S1) or CCMGE 628/12457 (Sues et al., 2015; Witmer, 2019). In these specimens, these openings lie at the bottom of the shapeless, wider depression limited by the prootic crest rostrally and the tuberal crest caudally. The condition is comparable in *S. musacchioi* (Martínez et al., 2016: fig. S1) and *G. brancai* (MB.R.2180.22.1-4; F Knoll, 2009, personal observations), suggesting that the conformation of the middle ear (tympanic) recess in FAM 03.064 and *L. pandafilandi* (recessed metotic cavity) is derived.

We previously associated a bilateral foramen at the base of the occipital condyle to the hypoglossal (XII) nerve (Díez Díaz et al., 2012: fig. 5C–F). CT scan data confirm this, but also reveal the presence of another hypoglossal (XII) canal about 8 mm more rostrally, which opens onto the caudal wall of the middle ear recess (Figs. 1 and 2). This rostral branch of the hypoglossal has a lesser diameter and extends in a more ventral direction than the caudal one. Only a caudal (i.e., one that shows up externally in a caudal situation with respect to the tuberal crest) hypoglossal branch was identified in *L. pandafilandi* (Knoll et al., 2013: figs 3A, D, S1, S2, S3), MPCM-HUE-1667 (Knoll et al., 2015: figs 4, 5C, D, S1) and *J. septentrionalis* (Wilson et al., 2009: fig. 7). However, a configuration similar to FAM 03.064 is observed in CCMGE 628/12457 (Sues et al., 2015: fig. 3A, C). This does not indicate close affinities between FAM 03.064 and CCMGE 628/12457 (see Discussion below) as the same configuration is also described in *G. brancai* (Janensch, 1935–1936: p. 260).

#### Endocranial vasculature

Only two vascular structures could be reconstructed digitally in FAM 03.064. Díez Díaz et al. (2012) recognized one little foramen in the orbital region as the outlet of an orbitocerebral vein, but this could not be traced digitally. A similar structure was identified in MPCM-HUE-1667, but was not reconstructed either (Knoll et al., 2015: p. 17). Although none is apparent on the endocast of *J. septentrionalis* (Wilson et al., 2009: fig. 7), it is not possible to say if a similar slender cerebral vasculature was present and the rostrolateral braincase region is poorly preserved in *L. pandafilandi* (Knoll et al., 2013: figs 1B, 2B, S1, S2, S3) and CCMGE 628/12457 (Sues et al., 2015: figs 2A, B, C; Witmer, 2019). The only venous structure that could be reconstructed in FAM 03.064 is a vein that emerges from a sinus that is dorsal to the trigeminal nerve in the dorsoventral axis, and leaves the endocranial cavity via the occipital plate (Figs. 1, 2A and 2C). This is part of the dorsal-head/caudal-middle-cerebral vein system, which is unnoticeable in *L. pandafilandi* (Knoll et al., 2013: figs 3A, B, C, S1, S2, S3), MPCM-HUE-1667 (Knoll et al., 2015: figs 4, 5A, B, D, S1) and *J. septentrionalis* (Wilson et al., 2009: fig. 7A). A caudal middle cerebral vein is present in CCMGE 628/12457 (Sues et al., 2015: figs 3A, B, D; Witmer, 2019), but it is different from that in FAM 03.064, notably because it orientates caudolaterally from the outset instead of extending laterally. In contrast, an approximately similarly orientated caudal middle cerebral vein is present in *B. reigi* (Paulina Carabajal, 2012: figs 1-3).

The cerebral carotid arteries are the main vascular structures that could be traced (Figs. 1, 2A and 2B). The two contralateral vessels are farther apart in FAM 03.064 and CCMGE 628/12457 (Sues et al., 2015; Witmer, 2019) than in MPCM-HUE-1667 (Knoll et al., 2015: fig. S1). In FAM 03.064 and MPCM-HUE-1667 (Knoll et al., 2015: fig. S1), they diverge by about 40° when seen in caudal view of the endocasts, and by about 70° in CCMGE 628/12457 (Sues et al., 2015; Witmer, 2019). A basilar artery is supposed to have originated from the caudal ramus of the carotid and run caudalward on the ventral surface of the brainstem. In FAM 03.064, no evidence of the basilar artery is detectable on the endocast, neither as a canal through the dorsum sellae between the pituitary and the hindbrain, as in CCMGE 628/12457 (Sues et al., 2015; Witmer, 2019) and *B. reigi* (Paulina Carabajal, 2012: fig. 3), for examples within Titanosauria, nor as a groove on the floor of the caudal portion of the braincase, as in the diplodocoid *Dicraeosaurus hansemanni* (MB.R.2378.1-5; F Knoll, 2009, personal observations). In FAM 03.064, the basal artery might have left the endocranial cavity to run on the ventral side of the neck of the occipital condyle in the groove noted by Díez Díaz et al. (2012: fig. 5C, F).

#### Inner ear

Both endosseous labyrinths could be reconstructed in FAM 03.064 (Figs. 2 and 3). They are arched medially, mimicking as they do the curvature of the internal surface of the braincase cavity at this place (i.e., the space constraints in this region of the braincase shape both the labyrinths and the portion of the main endocranial cavity between the two otic capsules).

**Figure 4:**
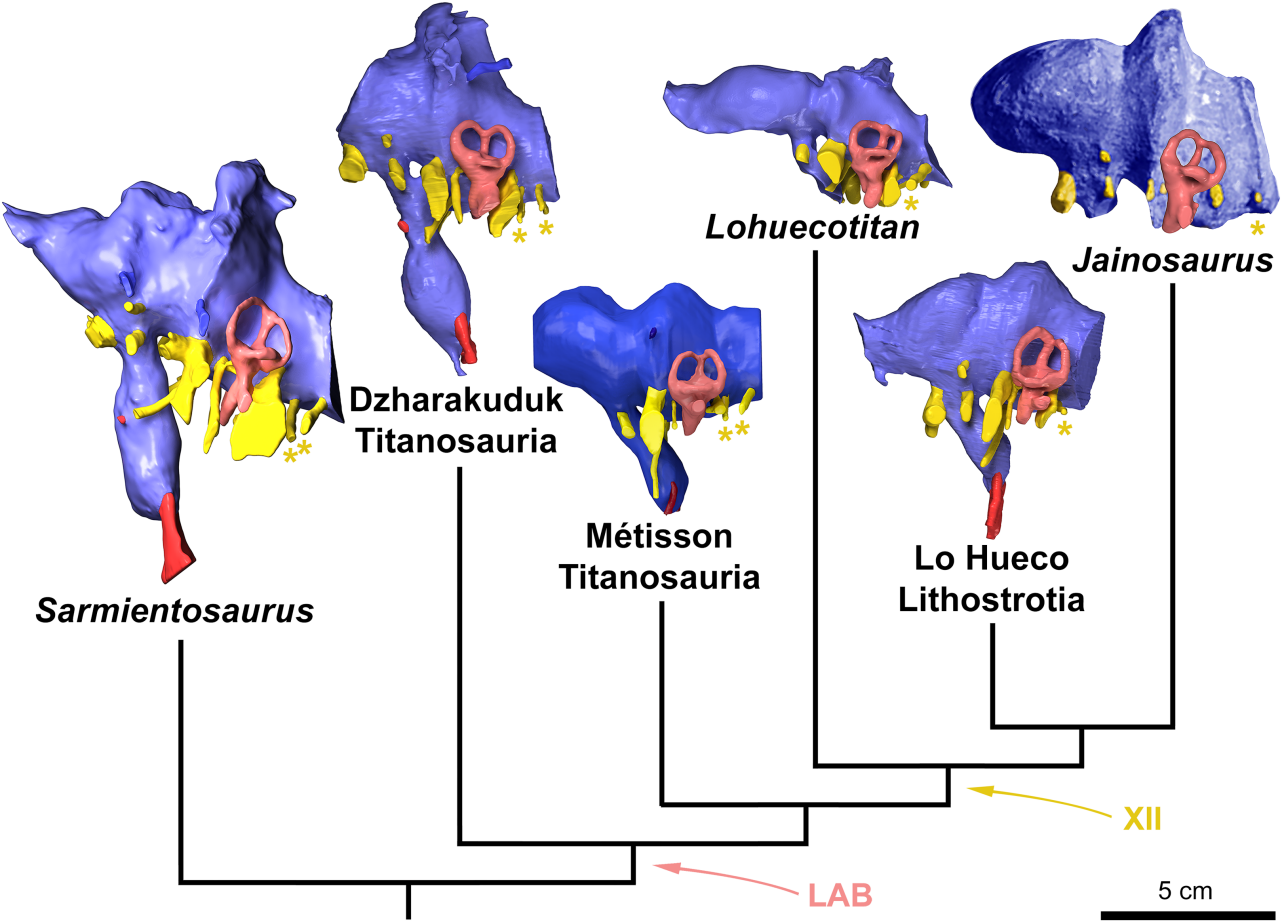
Endocasts of the most relevant sauropod taxa discussed in the text in a phylogenetic context. From left: *Sarmientosaurus musacchioi* (MDT-PV 2, from Martínez et al., 2016), indeterminate titanosaur from Uzbekistan (CCMGE 628/12457, from Sues et al., 2015), *Lohuecotitan pandafilandi* from Spain (MPCM-HUE-8741, right side mirrored, from Knoll et al., 2013), indeterminate titanosaur from Spain (MPCM-HUE-1667, from Knoll et al., 2015) and *Jainosaurus septentrionalis* (composite). In left lateral view. All are digital endocasts, save for *Jainosaurus septentrionalis*, which is a montage of a photograph of the physical endocast made from the lectotypic braincase (GSI K27/497, right side mirrored and colorized, from Wilson et al., 2009) combined with the digital reconstruction of the endosseous labyrinth from a different specimen referred to the same species (ISI R162, from Knoll et al., 2012). LAB and XII indicate synapomorphic reduction of the labyrinth of the inner ear (pink) and loss of one hypoglossal canal (asterisks) in the neurocranial wall, respectively. Note the trend towards a reduction of the dural membrane and associated venous channels, resulting in the uncluttered endocasts of most advanced titanosaurs.

The rostral and caudal semicircular canals are subequal in length (Fig. S1; Table 2). Indeed, the caudal semicircular canal of the left labyrinth appears to be slightly taller than the rostral semicircular canal, but the situation is reverse on the right side, implying suboptimal sensitivity or, in other words, relaxed selective constraints on gaze stabilization and other vestibular-controlled functions. Both vertical semicircular canals are significantly longer than the lateral semicircular canal (Fig. 3; Table 2). Titanosaurs were considered as being characterized by vertical semicircular canals of subequal lengths (Paulina Carabajal, 2012; Knoll et al., 2015). Indeed, CCMGE 628/12457, *L. pandafilandi*, MPCM-HUE-1667 and *J. septentrionalis* (ISI R162) have all relatively contracted vestibular regions (see Knoll et al., 2015: fig. 6). However, a more basal form, such as *S. musacchioi* (Martínez et al., 2016: figs 11, S1), shows a rostral semicircular canal that is intermediate in development between that of *G. brancai* (Knoll et al., 2012: fig. 6) and those of these more advanced lithostrotian titanosaurs (Fig. 4). The two vertical semicircular canals diverge at about 70° (Fig. 3C), which falls between the configurations observed in *L. pandafilandi* (Knoll et al., 2013: figs S1, S2, S3) and MPCM-HUE-1667 (Knoll et al., 2015: figs 7C, S1). The divergence angle of the two vertical semicircular canals is wider in both CCMGE 628/12457 (Sues et al., 2015: fig. 4B; Witmer, 2019) and *J. septentrionalis* (ISI R162; Sues et al., 2015: fig. 4E).

**Table 2:** Measurements of the endocast of the indeterminate titanosaurian braincase (FAM 03.064) from the Late Cretaceous of Fox-Amphoux-Métisson, France, and some of its components.

Component	Length (cm)	Width (cm)	Diameter (cm)	Volume (cm^3^)
Endocast	6.44	4.49	–	52
Cerebral hemispheres	2.58	3.68	–	21
Lagena (average)	1.00	0.62	–	0.3
Lateral semicircular canal (average)	–	–	0.34	–
Rostral semicircular canal (average)	–	–	0.52	–
Caudal semicircular canal (average)	–	–	0.55	–

The lagena is relatively short in FAM 03.064 (Figs. 3A and 3B; Table 2). The relative length of the lagena varies within advanced lithostrotians, such as CCMGE 628/12457 (Sues et al., 2015: fig. 4A, C; Witmer, 2019), *L. pandafilandi* (Knoll et al., 2013: figs 4, S1, S2, S3), MPCM-HUE-1667 (Knoll et al., 2015: figs 7A, B, S1) and *J. septentrionalis* (ISI R162; Sues et al., 2015: fig. 4D, F). Nonetheless, it is shorter than in more primitive forms such as *S. musacchioi* (Martínez et al., 2016: figs 11A, B, S1).

## Discussion

Considerable advances in our understanding of titanosaurian endocranial anatomy over the last few years have been paralleled by substantial progress in our perception of the topology of titanosaurian phylogeny (see in particular Sallam et al., 2018; Gorscak & O’Connor, 2019; Mannion et al., 2019a, 2019b), which had been particularly labile until then. As it turned out, the study of the osteology of FAM 03.064 through external observation did not provide insight regarding the phylogenetic position of the species to which it belongs within Titanosauria (Díez Díaz et al., 2012). Nevertheless, palaeoneurological information uncovered in the present work and evaluated in the light of recent thorough phylogenetic analyses of Titanosauria permits some clarification to be provided on this issue (Fig. 4). Naturally, not all palaeoneurological features are equally valuable indicators of phylogenetic relationships. For example, it seems that the lateral distance with which the abducens nerve makes its way past the pituitary is particularly prone to homoplasy. Indeed, in a variety of titanosaurs, such as MPCM-HUE-1667 (Knoll et al., 2015: figs 5A, B, C, S1), which is most likely more derived than FAM 03.064, but also CCMGE 628/12457 (Sues et al., 2015: fig. 3B, C, E; Witmer, 2019) and *S. musacchioi* (Martínez et al., 2016: figs 9C, S1), for instance, which are certainly more basal, the abducens nerve and the pituitary are farther apart than in FAM 03.064. Likewise, we do not place too much systematic importance either on the observed variations of angles between the planes of ipsilateral semicircular canals. First and foremost, these characters are deeply dependent on the degree of deformation of the specimens. We must also recall that the relative developments of the vestibular system are distinctly different from one another in braincases referred to *G. brancai* (Knoll et al., 2014), suggesting weak stabilizing selection on the vestibular labyrinth (which, in turn, might be related to relatively sluggish behaviour and/or little reliance on highly coordinated eye movements) in this and maybe many other sauropod species. This notwithstanding, sauropod braincases are not subject to the same selective pressures as the rest of the skeleton and as such palaeoneurology is doubtless a source of helpful further data for phylogenetic inferences (Balanoff, Bever & Ikejiri, 2010; Knoll et al., 2012; Bronzati, Benson & Rauhut, 2018).

A single bilateral hypoglossal canal was identified in the osteological description of FAM 03.064 (Díez Díaz et al., 2012). The CT data reveals that this was the caudal component of a pair of rami, the rostral constituent of which is very close to the metotic group (left side) or not clearly individualised from it (right side). A single hypoglossal root (XII) was considered characteristic of titanosaurs (Paulina Carabajal, 2012), but it rapidly turned out that several members of the group show two roots (see e.g., Sues et al., 2015; Martínez et al., 2016). As two hypoglossal roots are seen in the basal titanosauriforms *G. brancai* (Knoll & Schwarz-Wings, 2009), *E. holgeri* (Schmitt, Knoll & Tschopp, 2015) and indeed most sauropods, this represents the plesiomorphic condition. This character-state suggests that the titanosaur from Fox-Amphoux-Métisson is more primitive than the roughly coeval Lo Hueco lithostrotians and the distinctly more recent *J. septentrionalis* as well as, in fact, all the titanosaurs with a single hypoglossal rootlet on the endocast, such as *B. reigi* (Paulina Carabajal, 2012: figs 2A, 3), the derived non-saltasaurid *Diamantinasaurus matildae* (Poropat et al., 2016: fig. 5c, d) and the basalmost lithostrotian (clade specifier) *M. dixeyi* (Andrzejewski et al., 2019: figs 2A, C, S1). We suspect that the rostral hypoglossal canal coalesced with the cavum metoticum in a derived clade of titanosaurs resulting in an apparent single hypoglossal branch in the neurocranial endocasts of the taxa belonging to this lineage. Data are too deficient to trace a definite pattern across the phylogeny of titanosaurs. Nevertheless, the rostral hypoglossal canal is fairly equidistant from that shared by the glossopharyngeal and vagoaccesory nerves rostrally and the one for the caudal hypoglossal root caudally in *S. musacchioi* (Martínez et al., 2016: figs 9C, S1), whereas it is noticeably closer to the metotic group in a more derived form like the Dzharakuduk sauropod (Sues et al., 2015: fig. 3C; Witmer, 2019). Furthermore, even though this is not an analogous phenomenon, it should be remarked that an ontogenetical trend for the most rostral embryonic hypoglossal canal to unit with the vagal canal is known in sauropsids (Starck, 1979: pp. 16-17). The fact that the rostral hypoglossal appears to have merged with the metotic group on one side but not on the other might support an intermediate position of FAM 03.064 within the phylogeny of titanosaurs between taxonomic units such as CCMGE 628/12457 (showing metotic group and rostral hypoglossal well separated on both sides; Sues et al., 2015: fig. 3A, C; Witmer, 2019) basally and others such as *L. pandafilandi* (with no visible rostral hypoglossal on either side; Knoll et al., 2013: figs 3A, D, S1, S2, S3) apically.

The phylogenetic position of the Uzbek specimen CCMGE 628/12457 is especially relevant to the present work because FAM 03.064 appears more derived in virtually every aspect. The Bissekty Formation sauropod has been recently regarded as a possible non-lithostrotian titanosaur on the basis of the absence of definitive sauropod osteoderms among the numerous vertebrate remains collected from the original site, Dzharakuduk (Averianov & Sues, 2017: p. 189). Nevertheless, bones of sauropods are extremely rare at Dzharakuduk and similar localities (Nesov, 1995: p. 20), and titanosaurian osteoderms are generally scarcely found (D’Emic, Wilson & Chatterjee, 2009), even in sites in which these sauropods are common (see e.g., Bellevue in France, Le Loeuff et al., 1994, and Lo Hueco in Spain, Vidal, Ortega & Sanz, 2014). More importantly, the possession of osteoderms is at best an ambiguous synapomorphy of Lithostrotia (see e.g., D’Emic, 2012: table 5) and, very probably, not all members of this clade bore such bony structures (see Martínez et al., 2004). Averianov & Sues (2017: p. 192) noted that the Dzharakuduk sauropod presents similarities to *Dongyangosaurus sinensis* and *Baotianmansaurus henanensis*. According to Mannion et al. (2013: p. 158), *D. sinensis*, and possibly *B. henanensis* as well, would represent a titanosaur with close relationships to *Opisthocoelicaudia skarzynskii*. However, *D. sinensis* and *B. henanensis* were later recovered as non-lithostrotian titanosaurs (Mannion, Allain & Moine, 2017: fig. 39, 2019a: fig. 5, 2019b: fig. 42), more in line with the view of Averianov & Sues (2017: pp. 192–193). If we adopt a parsimonious approach and assume that the reduction in the number of hypoglossal roots to one occurred only once at the ancestral node of Lithostrotia (or even more basally, at some point along the branch leading from the basalmost node of Titanosauria to Lithostrotia) and no reversal happened thereafter, then the titanosaur from Dzharakuduk cannot be a member of Lithostrotia, nor can *S. musacchioi*, which also show two hypoglossal rami (Martínez et al., 2016: figs 9A, C, S1). Whether or not CCMGE 628/12457 and *S. musacchioi* belong in Lithostrotia, their respective phylogenetic position with one another and with regard to the Late Cretaceous titanosaurs from Eurasia and India that have been primarily considered in the present study can be reasonably determined, with some caution due to the impossibility of assessing individual variation (Fig. 4). The reduced inner ear labyrinth of the Dzharakuduk sauropod suggests that it is more derived than *S. musacchioi*, whereas the thickness and convolutedness of its dural membrane and the related development of the associated venous sinuses and emissary veins (see below) implies that it is less advanced than the titanosaur from Fox-Amphoux-Métisson and *a fortiori* those with a single hypoglossal branch.

The distinct narrow channel that runs from the facial foramen down to the ventral border of the parabasisphenoid of FAM 03.064 (as preserved—most of this bone is lacking; Díez Díaz et al., 2012: figs 4, 5E, F) may also carry some phylogenetic information. No such groove is visible on the braincase of the Lithostrotia indet. from Lo Hueco (Knoll et al., 2015), but a similar feature is present on a fragmentary neurocranial wall recently described and attributed to *Atsinganosaurus velauciensis* (Díez Díaz et al., 2018: fig. 2A). *A. velauciensis* is a titanosaur from the Campanian of Velaux-La Bastide Neuve (Garcia et al., 2010). Velaux-La Bastide Neuve is only about 70 km from Fox-Amphoux-Métisson and the sediments of the two localities have presumably deposited in a short temporal interval around the transition from the Middle Campanian to the Late Campanian (see Leleu, Ghienne & Manatschal, 2005; Cojan & Moreau, 2006). Recent phylogenetic results suggest that *Atsinganosaurus* is a more basal opisthocoelicaudiine than taxa such as *Lohuecotitan* and *Lirainosaurus* (Sallam et al., 2018: fig. 3; Gorscak & O’Connor, 2019: fig. 29). This may well hold true for the Fox-Amphoux-Métisson titanosaur as well.

The fact that the junction of the antotic and prootic crests constitutes the ventral border of the trigeminal foramen in FAM 03.064 makes this region of the lateral wall of the braincase similar to that in MPCM-HUE-1667 (Knoll et al., 2015: figs 3C, S1) but notably different from that in *G. brancai* (Janensch, 1935–1936: figs 2, 5), where these two crests converge much more ventrally. Titanosauriforms more derived than *G. brancai*, such as *M. dixeyi* (Andrzejewski et al., 2019: figs 1A, B, S1) and CCMGE 628/12457 (Sues et al., 2015; Witmer, 2019), also appear more primitive than FAM 03.064 in having these crests providing a space for the possible accommodation of a ventral branch of the trigeminal nerve.

As alluded to above, the level of development of the dorsal-head/caudal-middle-cerebral vein system has also a bearing on our appreciation of the phylogenetic position of FAM 03.064. This structure is particularly elaborated in *Spinophorosaurus nigerensis* (Knoll et al., 2012: figs 4A, B, D, S1, S2, S3), which is likely a basal sauropod (Holwerda & Pol, 2019). Generally speaking, it is well developed in sauropods (see e.g., Witmer et al., 2008), including non-titanosaurian titanosauriforms such as *G. brancai* (Knoll & Schwarz-Wings, 2009: figs 1, 2A, B, C, D) and *Tambatitanis amicitiae* (Saegusa & Ikeda, 2014: fig. 4F). However, it is unnoticeable in derived titanosaurs such as those from Lo Hueco (Knoll et al., 2013: figs 3A, B, C, S1, S2, S3; Knoll et al., 2015: figs 4, 5B, D, S1), *J. septentrionalis* (Wilson et al., 2009: fig. 7A) and possibly the other titanosaurs from India mentioned by Witmer et al. (2008: p. 76). Therefore, the degree of expansion of this complex on the endocast of the titanosaur from Fox-Amphoux-Métisson supports a position more basal within the phylogeny of Titanosauria than that of those Late Cretaceous taxa from Eurasia and India. On the other hand, the sauropod from Fox-Amphoux-Métisson appears closer to them than to the titanosaur from Uzbekistan (Sues et al., 2015: fig. 3A, B, D; Witmer, 2019) in the lack of prominent dural expansion on the dorsal surface of the hindbrain (Fig. 4).

Finally, we suggest that FAM 03.064 is not overly closely related to the only other titanosaur named from the uppermost Cretaceous of France for which neurocranial material is available, *Ampelosaurus atacis* (see Díez Díaz et al., 2012: p. 635). The latter appears closer to Spanish lithostrotians such as those of the Lo Hueco site, which presumably branched further from the base of Titanosauria than the Fox-Amphoux-Métisson sauropod did (Fig. 4). Palaeoneurological investigations into *A. atacis* would provide valuable data concerning the respective phylogenetic positions—and biogeographical history—of the titanosaurs from the latest Late Cretaceous of Var, Aude and Cuenca.

## Conclusion

The palaeoneurology of the titanosaurian braincase from the Campanian of Fox-Amphoux-Métisson reveals a number of characters of possible phylogenetic interest. These include the courses or number of canals followed by certain cranial nerves. The degree of development of the dural envelope and the associated venous channels in FAM 03.064 also appears to be indicative of its phylogenetic position within Titanosauria. Although not all the characters of FAM 03.064 may hint at a consistent phylogenetic position, the discrepancies are reasonable. This specimen appears to be from a species distinctly more advanced than the titanosaur from the lower Upper Cretaceous of Dzharakuduk (Sues et al., 2015), which exhibits a developed dura, but possibly less derived than those from the upper Upper Cretaceous of Lo Hueco (Knoll et al., 2013, 2015), which have only a single bilateral hypoglossal root. However, as the pattern of modification of the neurocranium within the evolution of Titanosauria is still poorly known, a number of features such as the level of thickness and morphological complexity of dural expansions in this group of sauropods might be more plastic than here considered. The growing number of studies such as this one in concert with an ever-consolidating phylogenetic framework will gradually but quite certainly enable a more positive picture of neurocranial changes during titanosaurian evolution to emerge.

## Supplemental Information

10.7717/peerj.7991/supp-1 Supplemental Information 1 Interactive three-dimensional digital visualization of the braincase, cranial endocast and associated soft tissues structures of the indeterminate titanosaurian specimen (FAM 03.064) from the Late Cretaceous of Fox-Amphoux-Métisson, France.

## Institutional abbreviations

CCMGE: Chernyshev’s Central Museum of Geological Exploration, Saint Petersburg, Russia
FAM: Mairie de Fox-Amphoux, Fox-Amphoux, France
GSI: Geological Survey of India, Kolkata, India
ISI: Indian Statistical Institute, Kolkata, India
MB.R.: Collection of fossil Reptilia, Museum für Naturkunde, Berlin, Germany
MDT-PV: Museo Desiderio Torres-Paleovertebrados, Sarmiento, Argentina
MPCM: Museo de Paleontología de Castilla-La Mancha, Cuenca, Spain

## Additional Information and Declarations

### Data Availability

The following information was supplied regarding data availability: The CT scan data and Avizo label files are available at: https://www.morphosource.org/Detail/ProjectDetail/Show/project_id/851.
