# Supplementary figures and images for: First palaeoneurological study of a sauropod dinosaur from France and its phylogenetic significance
Source: PeerJ. 2019 Nov 18;7:e7991. doi: 10.7717/peerj.7991 (PMC6871212; doi:10.7717/peerj.7991)

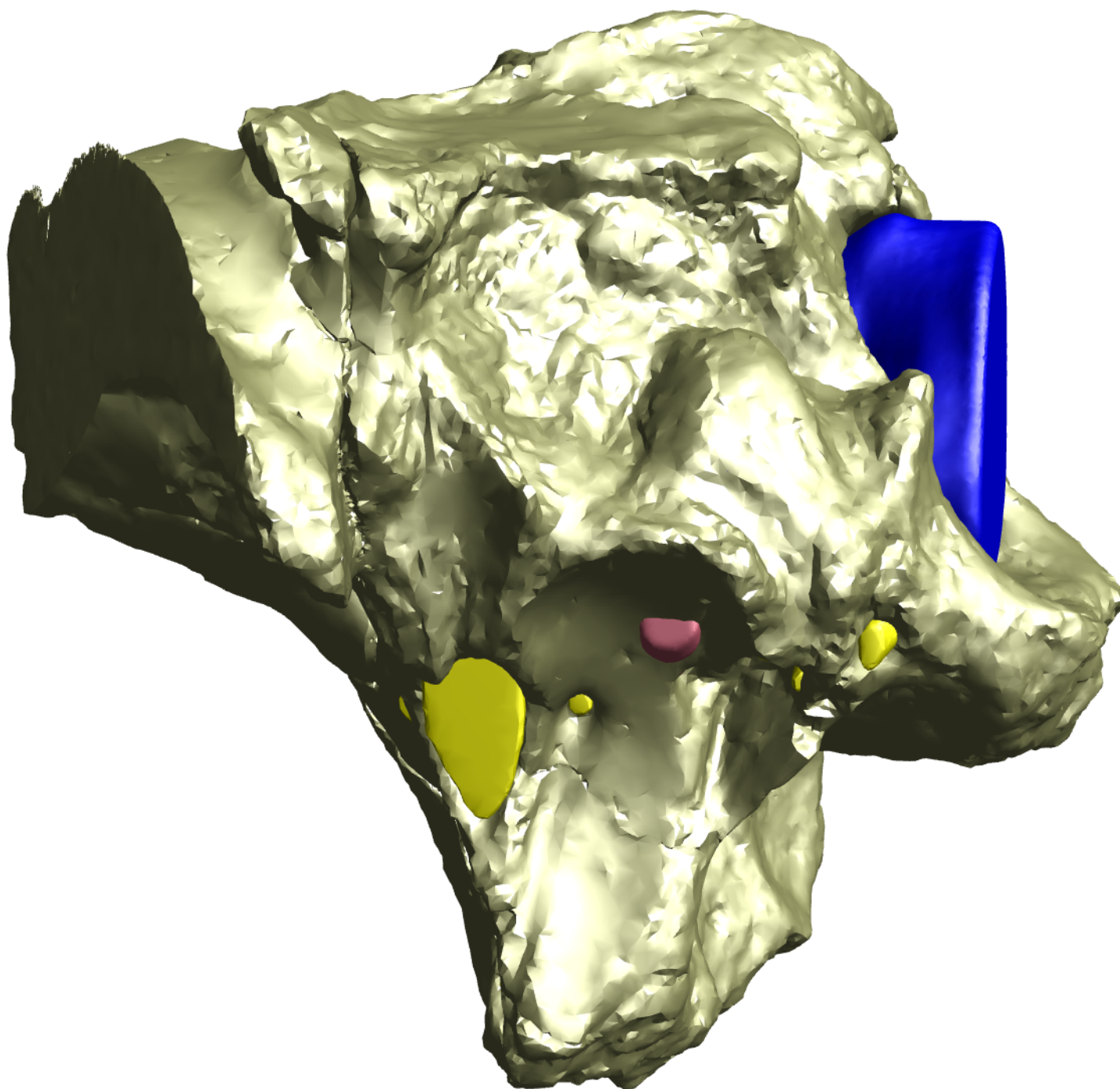

Supplement: Supplemental Information 1 [file peerj-07-7991-s001.pdf]
